# Free and bound phenolic profiles of *Radix Puerariae Thomsonii* from different growing regions and their bioactivities

**DOI:** 10.1016/j.fochx.2024.101355

**Published:** 2024-04-05

**Authors:** Weixin Li, Mingwei Zhang, Xuchao Jia, Min Zhang, Yanxia Chen, Lihong Dong, Fei Huang, Qin Ma, Dong Zhao, Ruifen Zhang

**Affiliations:** a State Key Laboratory of Food Nutrition and Safety, College of Food Science and Engineering, Tianjin University of Science and Technology, Tianjin 300457, PR China; b Sericultural & Agri-Food Research Institute Guangdong Academy of Agricultural Sciences/Key Laboratory of Functional Foods, Ministry of Agriculture/Guangdong Key Laboratory of Agricultural Products Processing, Guangzhou 510610, PR China

**Keywords:** *Radix puerariae thomsonii*, Phenolic composition, Antioxidant activity, Alcohol metabolizing enzymes, Carbohydrate hydrolyzing enzymes

## Abstract

The free and bound phenolic profiles and their bioactivities of *radix puerariae thomsonii* (RPT) cultivars from 7 growing regions in China were investigated. Total phenolic and flavonoid contents were from 148.71 to 435.32 mg gallic acid equivalents /100 g dry weight and 561.93 to 826.11 mg catechin equivalents /100 g dry weight, respectively, with 20.64–38.28% and 32.77–47.29% contribution from bound fractions. Sixteen phenolic compounds were detected in RPTs. Bound fractions contributed 28.15–70.84% to the total antioxidant activities. The cultivars from Qiannan and Guangzhou showed much higher regulatory effects on carbohydrate hydrolyzing enzymes and alcohol metabolizing enzymes than the other cultivars. The bound fractions exhibited equivalent EC_50_ values for alcohol metabolizing enzymes and IC_50_ values for carbohydrate hydrolyzing enzymes to the free fractions in RPT cultivars. Therefore, bound phenolics significantly contributed to the potential health benefits of RPT. The results provided information for the utilization of RPT for health promoting purpose.

## Highlights

• Phenolic content of *Radix Puerariae Thomsonii* (RPT) showed regional differences.
• Bound phenolics contributed a lot to the content and activity of RPT cultivars.
• Four phenolics including puerarin derivative A were firstly found in bound form.

## 1 Introduction

*Radix puerariae* (RP) is an important medicinal plant in traditional oriental medicine having many healthy effects, including antialcoholic, hepatoprotective, and cardioprotective activities (Jin, Son, Min, Jung, & Choi, 2012). Phenolics and flavonoids are considered the predominant components accounting for the health benefits of RP. *Radix puerariae thomsonii* (RPT) and *radix puerariae lobata* (RPL) are the most representative varieties of RP, widely distributed in Asia, North, and South America (Jin et al., 2012). RPL is usually used as medicine for its high contents of flavonoids, while RPT is commonly consumed as food in southern China (Wong, Razmovski-Naumovski, Li, Li, & Chan, 2015). As an important resource for functional foods, the planting areas of RPT have been expanding in China in the past 20 years. It has been planted in large scale in southeast and southwest of China, including Guangdong, Guangxi, Guizhou, Jiangxi, and Hunan provinces (Liu et al., 2021). As a vegetatively propagated crop, RPT has been cultivated by local farmers for generations to develop cultivars well-suited to the local climate and environment (Fu et al., 2023). Generally speaking, after long-term cultivation, crops in various growing regions could gradually form local advantageous cultivars, which often have certain genetic differences from the cultivars in the other regions. This phenomenon was commonly found in the major vegetatively propagated crops such as potato (Wang et al., 2019), sweet potato (Behera et al., 2024; Minemba, Gleeson, Veneklaas, & Ryan, 2019), yam (Dwivedi et al., 2023), and garlic (Parreño et al., 2023). However, the genotypes of these local cultivar RPTs were not identified by specialized agencies. The RPTs from different growing regions are usually considered as local cultivars.

Most existing studies on RP were focused on RPL, which was consumed as medicine rather than food. The RPLs collected from different regions of North Korea showed significantly different flavonoid profiles (Mun & Mun, 2015). However, available data on the regional differences in phenolic and flavonoid profiles of RPTs are limited. The main flavonoids in RPT have been identified as isoflavones, including puerarin, daidzin, genistin, ononin, and their aglycones (Mocan et al., 2018). Van Hung and Morita (2007) found that 3 commercial RPL starch products produced in Vietnam, Japan, and South Korea had different phenolic composition and contents. The differences in RPL cultivars used for starch production and their various growing environment were both responsible for such differences. Three different local cultivars of RPT cultivated in the same regions also had big differences in the contents of puerarin (5.3–20.3 mg/g dry weight, DW), daidzin (0.95–4.7 mg/g DW), and daidzein (0.33–2.5 mg/g DW), indicating the phenolic contents in RPT had big cultivar differences (Zhou, Wang, & Yan, 2006). In the main RPT growing provinces of China, the environmental factors, including altitude, annual precipitation, intensity of UV radiation, and soil fertility were all greatly different. Furthermore, local cultivars cultivated in different regions might have various genotypes. Therefore, we speculated that there might be significant regional differences in phenolic profiles of RPTs cultivated in different regions of China.

It is well known that phenolics exist in plants as free and bound forms. >20% of phenolics in fruits and vegetables usually occur in bound form (Zhang, Zhang, Li, Deng, & Tsao, 2020). However, bound phenolics in RPT were not involved in previous studies, which might result in underestimation of the total phenolic content and their bioactivities. The hepatoprotective and antidiabetic activities of RP had been reported previously, which was partly attributed to the regulatory effects of phenolics in RP on carbohydrate hydrolyzing enzymes and alcohol metabolizing enzymes (Keung & Vallee, 1998; Wang et al., 2017). However, the differences in regulatory effects of RPTs from different growing regions on the metabolic enzymes remain unknown.

Therefore, RPTs from 7 representative growing regions of China were analyzed in this study, aiming at (1) elucidating the free and bound phenolic profiles of RPTs and their regional differences; (2) investigating the antioxidant and enzyme regulating activities of phenolics in RPTs from different growing regions. The results could provide useful information for screening functional food raw material with high health promoting potential and guiding the nutrition-oriented processing of RPT.

## 2 Materials and methods

### 2.1 Preparation of RPT samples

Fresh roots of RPT samples from 7 main growing regions in China were collected from local agricultural production cooperatives during the period from December 2021 to January 2022 (the harvest season of RPTs). To ensure the representativeness of the samples, the RPT for each region was from 3 to 4 different farmers. The RPTs were named using the acronyms of their growing regions, which were GDGZ-RPT (Guangzhou, Guangdong), GDFS-RPT (Foshan, Guangdong), GDSG-RPT (Shaoguan, Guangdong), GXWZ-RPT (Wuzhou, Guangxi), YNQJ-RPT (Qujing, Yunnan), GZQN-RPT (Qiannan, Guizhou), JXGZ-RPT (Ganzhou, Jiangxi). The pictures of 7 RPTs are shown in Fig. S1. The RPT samples were peeled, sliced and dried at 50 °C. The dried RPT were crushed into powder, completely passed through a 60-mesh sieve, and stored at −18 °C until further use.

### 2.2 Chemical and reagents

Folin-Ciocalteu's reagent, 6-hydroxy-2,5,7,8-tetramethylchroman-2-carboxylic acid (Trolox), 2,2′-Azobis(2-methylpropionamidine) dihydrochloride (AAPH), fluorescein sodium salt, 2′,7′-dichlorofluorescin diacetate (DCFH-DA), Tris(hydroxymethyl)methyl aminomethane, β-Nicotinamide Adenine Dinucleotide (β-NAD), β-Mercatoethanol (β-Me) and α-D-glucopyranoside (*p*NPG) were obtained from Sigma-Aldrich (St. Louis, MO, USA). The enzymes used, including alcohol dehydrogenase (ADH) from *saccharomyces cerevisiae*, potassium-activated aldehyde dehydrogenase (ALDH) from yeast, α-glucosidase from *saccharomyces cerevisiae*, and α-amylase from porcine pancreas were all purchased from Sigma-Aldrich (St. Louis, MO, USA). Standard phenolic compounds including 3′-hydroxypuerarin, 6″-*O*-xylosidepuerarin, puerarin, mirificin, 3″-methyoxypuerarin, daidzin, rutin, ferulic acid, genistin, phlorizin, ononin, daidzein, genistein, quercitrin, and *p*-coumaric acid were obtained from Yuanye Biotechnology Co., Ltd. (Shanghai, China). HPLC-grade acetic acid and acetonitrile were purchased from Fisher Scientific (Waltham, MA, USA). All other reagents used were of analytical grade.

### 2.3 Extraction of free phenolics

Free phenolics were extracted by the method described previously (Zhang, Zhang, Zhang, & Liu, 2010). Briefly, 1.0 g of RPT powder was blended with 40 mL of precooled 80% acetone and homogenized using IKA T25-Ultra-Turrax (Stauffen, Germany) at 1.0 × 10^4^ rpm for 10 min in an ice bath. The homogenate was centrifuged and the sediment was extracted twice under the same conditions. The pooled supernatants were evaporated under vacuum at 45 °C, and reconstituted to 10 mL with MeOH to get the free phenolic extracts. The extracts were stored at −20 °C for further analysis.

### 2.4 Extraction of bound phenolics

Bound phenolics were extracted according to the previous method (Zhang et al., 2010). The residue from free phenolic extraction was hydrolyzed using 2.0 mol/L NaOH (1:20, *w/v*) at room temperature for 1 h with continuous shaking under a N_2_ atmosphere. The mixture was then neutralized with 6.0 mol/L HCl and extracted 5 times with equal volume ethyl acetate. The pooled ethyl acetate fractions were evaporated at 45 °C to dry and reconstituted with MeOH to 10 mL. The bound phenolic extracts were stored at −20 °C until use.

### 2.5 Determination of total phenolic and flavonoid contents in RPT

#### 2.5.1 Total phenolic content (TPC)

The TPC was analyzed using the Folin-Ciocalteu (FC) assay as previously described (Zhang et al., 2019). Gallic acid was employed as the standard. The results were expressed as milligrams of gallic acid equivalents (GAE) /100 g dry weight (DW) of sample.

#### 2.5.2 Total flavonoid content (TFC)

The TFC was determined through sodium borohydride/chloranil (SBC) assay using catechin as standard and expressed as milligrams of catechin equivalents (CE) /100 g DW of sample according to the method described by He, Liu, and Liu (2008).

### 2.6 Determination of the composition and content of phenolic compounds

Phenolic compounds were determined according to a previously reported HPLC method (Zhang et al., 2019) with some modification. The HPLC system (Agilent, Waldbronn, Germany) was equipped with a DAD detector and a Zorbax SB-C18 analytical column (250 × 4.6 mm, i.d., 5 μm, Palo Alto, CA, USA). The mobile phase consisted of solution A (0.4% aqueous acetic acid) and solution B (acetonitrile) at a flow rate of 1.0 mL/min. The gradient elution program was performed as follows: solution B: 0–25 min, 5–25%; 25–30 min, 25–35%; 30–40 min, 35–60%. The column temperature was maintained at 30 °C, and the injection volume was 20 μL. All the samples were filtered through a 0.22 μm membrane filter (Millipore, Billerica, MA, USA) before analysis. The detection wavelength was set to 280 nm. The monomer phenolic compounds in RPT were identified by comparing their retention times and UV spectral data with the authentic standards of the previously reported compounds in RPT (Wu et al., 2019). Quantitative analysis was carried out using our previous method (Feng et al., 2021) with a nano-UHPLC system equipped with the Triple-TOF™ 5600+ mass spectrometer (SCIEX, Framingham, MA, USA) operating in both negative-ion (ESI-) and positive-ion (ESI+) mode. The operation parameters were set as follows: source voltage, ESI- (4.5 kV) and ESI+ (5.5 kV); capillary temperature, 300 °C; sheath gas flow, 30 arbitrary units and auxiliary gas flow, 5 arbitrary units. The mass range for ESI experiments was set *m*/*z* from 100 to 1000. Data processing was conducted using Peakview 2.2 and Multiquant 3.0.3 (SCIEX, Framingham, MA, USA).

### 2.7 Estimation of antioxidant capacity of RPT

#### 2.7.1 Antioxidant activity determined by oxygen radical antioxidant capacity (ORAC) assay

The ORAC analysis was carried out using a previously established method (Zhang et al., 2010). The determination was performed with the black-walled 96-well plates. Twenty microliter appropriately diluted extracts or Trolox with concentrations of 6.25 to 50 μmol/L together with 200 μL fluorescein (0.96 μmol/L) were added into each well. In the blank wells, 20 μL PBS (75 mmol/L, pH 7.4) was used instead of the extracts. After a 20-min incubation at 37 °C, 20 μL AAPH (119 μmol/L) was added to each well. The fluorescence intensity was measured using a microplate reader (Tecan Infinite M200PRO, Männedorf, Switzerland) at excitation/emission wavelengths of 485/538 nm for 35 cycles. The ORAC activity was expressed as μmol Trolox equivalents (TE)/ g DW of the RPT sample.

#### 2.7.2 Antioxidant activity determined by peroxyl radical scavenging capacity (PSC) assay

The PSC assay was performed following a previously reported method (Feng et al., 2021). The extracts and Trolox were diluted with 75 mmol/L PBS (pH 7.4). Then, 100 μL of diluted extracts was mixed with equivalent volume of DCFH solution and 50 μL of 200 mmol/L AAPH. DCFH solution was prepared by hydrolyzing 80 μL of 2.48 mmol/L DCFH-DA with 900 μL of 1.0 mmol/L KOH for 3–5 min to remove the diacetate. During the 40-min reaction, the fluorescence intensity was measured every 2 min at excitation/emission wavelengths of 485/538 nm. The PSC activity was expressed as μmol TE/g DW of the RPT sample.

### 2.8 Determination of the activation of ADH and ALDH by RPT

#### 2.8.1 ADH activation assay

ADH activation activity was determined by monitoring the formation of NADH at 340 nm using a modified method described by Keung and Vallee (1998). The reaction mixture consisting of 1.5 mL of 1.0 mol/L Tris-HCl buffer (pH 8.8), 1.0 mL of 20 mmol/L β-NAD, 0.5 mL of 50% ethanol (*v/v*), and 0.1 mL of RPT extracts diluted to different concentrations. Distilled water was used instead of the phenolic solution as a blank control. After being fully mixed, the mixture was pre-incubated at 30 °C for 5 min, followed by the addition of 0.1 mL of 0.5 U/mL ADH. The absorbance at 340 nm was measured every 30s for 5 min using a TU-1900 ultraviolet spectrophotometer (Beijing General Analytical Instrument Co., Ltd., Beijing, China). The effects of the extracts on ADH activation were quantified by the EC_50_, which represents the concentration at which 50% activation of the enzyme was achieved. EC_50_ was calculated by a linear regression probit model, as measured by activation rate to the base 10 logarithm of the extracts concentrations. The probit model parameters and EC_50_ values were obtained using SPSS statistic software (version 20.0). The activation rate of ADH was calculated as follows:

(1) $$\text{Activation rate}\;\left(\%\right)=\left(\frac{A_{1}-A_{0}}{A_{0}}\right)\times 100$$

where A_1_ represents the absorbance of the sample that contained extracts and A_0_ represents the absorbance of the blank control.

#### 2.8.2 ALDH activation assay

The ALDH activation activity was determined according to previously described method (Choi et al., 2009). The reaction mixture contained 0.1 mL of extracts at varying concentrations (with distilled water in the blank control), 0.1 mL of acetaldehyde (1.0 mol/L), 0.1 mL of β-NAD (20 mmol/L), 0.1 mL of KCl (3.0 mol/L), 1.6 mL of Tris-HCl buffer (1.0 mol/L, pH 8.0), and 0.1 mL of β-Me (0.33 mol/L). The mixture was incubated at 30 °C for 5 min. After being added 0.1 mL of 0.5 U/mL ALDH solution, the absorbance at 340 nm was recorded every 30s for 5 min by a TU-1900 ultraviolet spectrophotometer. The activation rate of ALDH was calculated in a similar manner to that of ADH. The activation effects of the tested samples were expressed as EC_50_, which was calculated using the method mentioned above.

### 2.9 Determination of the inhibition of RPT to α-glucosidase and α-amylase

#### 2.9.1 α-Glucosidase inhibition assay

The α-glucosidase inhibitory activity was determined following a previously reported method (Tadera, Minami, Takamatsu, & Matsuoka, 2006). In short, all extracts were diluted to concentrations ranging from 100 to 2000 mg DW/mL with PBS (0.1 mol/L, pH 6.8). Eighty microliters PBS, 20 μL extracts at different concentrations and 20 μL α-glucosidase (0.1 U/mL) were added to 96-well plates successively. After 15-min incubation at 37 °C, 20 μL *p*NPG (10 mmol/L) was added and the mixture was then incubated for 20 min at 37 °C. Finally, 100 μL Na_2_CO_3_ (0.2 mol/L) was added to terminate the reaction. The absorbance was measured at 405 nm using microplate reader. The inhibitory effects of the tested samples were expressed as IC_50_, representing the concentration at which 50% inhibition of the enzyme activity was observed. IC_50_ was calculated using the same method as EC_50_. The α-glucosidase inhibition rate (%) was calculated based on the following equation:

(2) $$\text{Inhibition rate}\;\left(\%\right)=\left(1-\frac{{\text{A}}_{\text{sample}}-{\text{A}}_{\text{blank}}}{{\text{A}}_{\text{control}}-{\text{A}}_{\text{background}}}\right)\times 100$$

where A_sample_, A_blank_, A_control_, and A_background_ represent the absorbance of the extracts (containing samples, PBS, and enzyme), the blank (containing samples and PBS), the control (containing PBS and enzyme), and the background (containing PBS buffer only), respectively.

#### 2.9.2 α-Amylase inhibition assay

The method reported by Tadera et al. (2006) was used for testing α-amylase inhibitory activity. Forty microliters of extracts were mixed with equal volume of α-amylase (0.5 U/mL) in a 96-well plate, and incubated at 37 °C for 5 min. Then 40 μL soluble starch (1.0 mg/mL) was added to each well. The reaction was terminated by adding 20 μL HCl (1.0 mol/L). Subsequently, 60 μL iodine reagent (5.0 mmol/L) was added and the absorbances were measured at 650 nm. The inhibitory effects of the extracts were expressed as IC_50_, while the calculation method for α-amylase inhibition rate was consistent with that of α-glucosidase.

### 2.10 Statistical analysis

All tests were repeated thrice. Results were represented by mean ± standard deviation (SD). Differences among RPTs from different regions were analyzed using one-way ANOVA followed by Duncan test. Statistical analyses were performed using IBM SPSS Statistics Version 20.0. A *p*-value <0.05 was considered statistically significant.

## 3 Results and discussion

### 3.1 Total contents of phenolics and flavonoids in RPTs

**Fig. 1:**
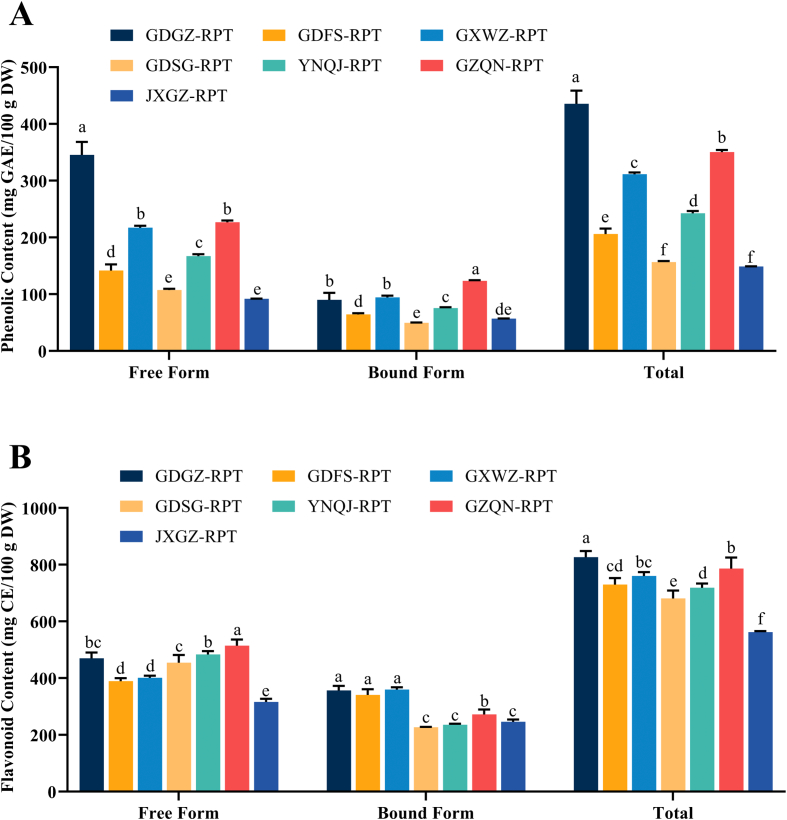
Total phenolic (A) and flavonoid (B) contents of RPTs from 7 growing regions. Bars with no common letters in the same group are significantly different (*p* < 0.05). GAE: gallic acid equivalents; CE: catechin equivalents; DW: dry weight; RPT: *radix puerariae thomsonii*.

The free, bound and total phenolic and flavonoid contents of RPTs from 7 different growing regions are presented in Fig. 1. The free and bound phenolic contents (Fig. 1A) ranged from 91.79 (JXGZ-RPT) to 345.47 (GDGZ-RPT) mg GAE/100 g DW and 49.24 (GDSG-RPT) to 123.44 (GZQN-RPT) mg GAE/100 g DW, respectively. Free phenolics accounted for 61.72% to 79.36% of TPC. GDGZ-RPT contained the highest TPC (435.32 mg GAE/100 g DW), followed by GZQN-RPT, GXWZ-RPT, YNQJ-RPT, and GDFS-RPT. The coefficient of variation (*CV*) of TPC in determined RPTs was 37.32%, indicating that the phenolic contents of RPT had significant regional variation. As the results showed in Fig. 1B, the free and bound flavonoid contents of 7 RPTs ranged from 315.84 (JXGZ-RPT) to 514.13 (GDGZ-RPT) and from 226.71 (GDSG-RPT) to 359.53 (GXWZ-RPT) mg CE/100 g DW, respectively. The percentages of free fractions to the total content were from 52.71% (GXWZ-RPT) to 67.23% (YNQJ-RPT). GDGZ-RPT had the highest TFC (826.11 mg CE/100 g DW), followed by GZQN-RPT, GXWZ-RPT, GDFS-RPT, and YNQJ-RPT, among which GXWZ-RPT showed similar TFC level to GZQN-RPT and GDFS-RPT (*p* > 0.05). JXGZ-RPT contained the least TFC (561.93 mg CE/100 g DW) among all the 7 determined RPTs (*p* < 0.05). To the best of our knowledge, this is the first instance in which the contents of bound phenolics and flavonoids in RPTs were determined. The contents of bound phenolics and flavonoids accounted for 20.64–38.28% and 32.77–47.29% of the total contents, respectively. Evidently, disregarding bound fraction would lead to a substantial underestimation of the total phenolic contents in RPTs. The TPC of the determined RPTs were lower than most vegetables previously reported by Lin and Tang (2007), such as red onion I (310.8 mg GAE/100 g fresh weight (FW)), ceylon spinach (269.0 mg GAE/100 g FW), and beetroot (257.2 mg GAE/100 g FW) when the moisture contents in RPTs (56.93–65.74%) were taken into consideration. But the RPTs tested in this study had much higher TFC than previously reported fruits (Chang et al., 2018; Deng et al., 2022) such as litchi (39.4 to 129.8 CE/100 g FW), apple (35.7 to 46.8 CE/100 g FW), and strawberry (46.2 to 70.5 CE/100 g FW).

Growth environment plays a crucial role in influencing the accumulation of secondary metabolites in plants. The TPC and TFC of 7 RPTs in this study showed notable regional differences, which might be closely related to local climate, temperature, and soil fertility. Among all the samples analyzed, GDGZ-RPT, cultivated in Guangdong province, and GZQN-RPT, cultivated in Guizhou province, had the highest and second highest TPC and TFC. The high temperature and humidity in Guangdong province, the highland environment and infertile soil in Guizhou province, along with the intense UV radiation in both regions, might serve as stress factors stimulating the production of secondary metabolites in RPTs. As reported by Qi et al. (2016), UV-B exposure could increase the activity of the key enzyme for phenolic synthesis, phenylalnine ammonialyase (PAL), and significantly enhance the contents of phenolics in soybean sprouts. In addition, the cultivation conditions of RPTs such as water and fertilizer could differ, which also contribute to the variation of their phytochemical contents.

However, the environmental factors alone could not entirely explain the regional differences in TPC and TFC among RPTs. The RPTs cultivated in different regions were predominantly local cultivars. As a crop propagated vegetatively, these local cultivars have been grown in their respective regions year after year. Despite a lack of genotype information of RPTs, different local cultivars were usually considered to have various genotypes (Wang et al., 2019). Therefore, genetic variation could also contribute to accounting for the regional differences in phenolic contents among RPTs. As shown in this study, Foshan city is adjacent to Guangzhou city, yet GDGZ-RPT, the RPT cultivated in Guangzhou, exhibited twice as much TPC as GDFS-RPT, the RPT cultivated in Foshan. Such substantial differences between 2 RPTs could be attributed, at least partly, to their different genotypes. Additionally, Chen, Cai, and Xu (2017) found that the free phenolic content of RPT cultivated in Zhuhai (a city adjacent to Guangzhou and Foshan) was 1.67 mg GAE/g DW, equivalent to that of GDFS-RPT as determined in our study. However, the total content of flavonoids of the Zhuhai local cultivar (0.30 mg CE/g DW) was far lower than those of all the RPTs determined in our study. Apart from genotype differences, the discrepancy in TFC was also due to the different determination methods used. Aluminum chloride colorimetric method was most commonly used for TFC determination, as seen in the study of Chen, Cai, and Xu (2017). However, it has been found that this approach could not detect all types of flavonoids, leading to a substantial underestimation of the total contents (Mammen & Daniel, 2012). In our current study, TFC was determined using the SBC assay, which has been proven to be highly accurate and specific to flavonoids (He et al., 2008).

### 3.2 Phenolic compositions and contents in RPTs

**Fig. 2:**
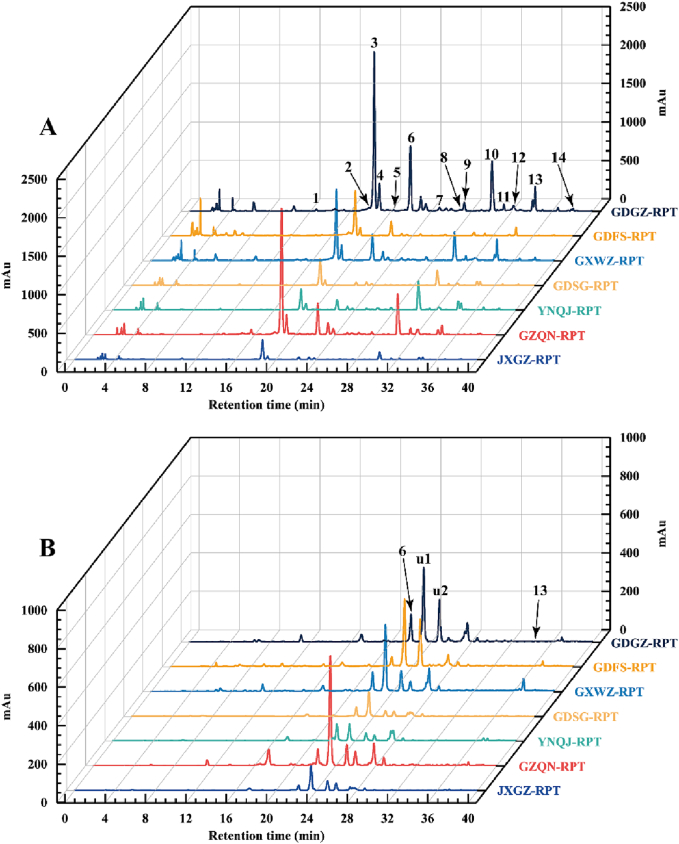
HPLC profile of free(A) and bound (B) phenolic of RPTs from 7 growing regions. Peaks 1, 3′-hydroxypuerarin; 2, 6″-*O*-xylosidepuerarin; 3, puerarin; 4, mirificin; 5, 3′-methoxypuerarin; 6, daidzin; 7, rutin; 8, ferulic acid; 9, genistin; 10, quercitrin; 11, phlorizin; 12, ononin; 13, daidzein; 14, genistein; u1, puerarin derivative A; u2, *p*-coumaric acid. RPT: *radix puerariae thomsonii*.

HPLC profiles of free and bound phenolics of RPTs from 7 growing regions are shown in Fig. 2. The compositions and contents of individual phenolic compounds in both free and bound forms of various RPTs are presented in Table 1. Sixteen phenolic compounds were detected in different RPTs, including 11 isoflavones (daidzin, daidzein, genistin, genistein, ononin, puerarin and its derivatives: 3′-hydroxypuerarin, 6″-*O*-xylosidepuerarin, mirificin, 3′-methoxypuerarin, and puerarin derivative A), 3 flavones (rutin, quercitrin, and phlorizin), and 2 phenolic acids (ferulic acid and *p*-coumaric acid). Among them, daidzin and daidzein were present in both free and bound forms, while puerarin derivative A and *p*-coumaric acid were only detected in bound form. The other 12 compounds were found in free form.

**Table 1:** Profiles of free and bound phenolics of RPTs from different growing regions (μg/g dry weight).

Compounds	RPTs	Free	Bound	Total
3′-hydroxypuerarin	GDGZ-RPT	79.84 ± 7.24^c⁎^ (100)^⁎⁎^	ND	79.84 ± 7.24^c^
	GDFS-RPT	92.74 ± 1.95^b^ (100)	ND	92.74 ± 1.95^b^
	GXWZ-RPT	142.49 ± 3.34^a^ (100)	ND	142.49 ± 3.34^a^
	GDSG-RPT	41.78 ± 0.85^d^ (100)	ND	41.78 ± 0.85^d^
	YNQJ-RPT	11.54 ± 0.92^e^ (100)	ND	11.54 ± 0.92^e^
	GZQN-RPT	44.44 ± 7.83^d^ (100)	ND	44.44 ± 7.83^d^
	JXGZ-RPT	35.01 ± 3.15^d^ (100)	ND	35.01 ± 3.15^d^
6″-*O*-xylosidepuerarin	GDGZ-RPT	136.17 ± 6.13^de^ (100)	ND	136.17 ± 6.13^de^
	GDFS-RPT	127.99 ± 5.76^de^ (100)	ND	127.99 ± 5.76^de^
	GXWZ-RPT	152.65 ± 3.93^d^ (100)	ND	152.65 ± 3.93^d^
	GDSG-RPT	359.67 ± 24.94^b^ (100)	ND	359.67 ± 24.94^b^
	YNQJ-RPT	108.67 ± 1.66^e^ (100)	ND	108.67 ± 1.66^e^
	GZQN-RPT	652.26 ± 12.53^a^ (100)	ND	652.26 ± 12.53^a^
	JXGZ-RPT	195.98 ± 19.37^c^ (100)	ND	195.98 ± 19.37^c^
puerarin	GDGZ-RPT	1340.60 ± 84.44^a^ (100)	ND	1340.6 ± 84.44^a^
	GDFS-RPT	775.10 ± 1.17^d^ (100)	ND	775.1 ± 1.17^d^
	GXWZ-RPT	912.74 ± 0.21^c^ (100)	ND	912.74 ± 0.21^c^
	GDSG-RPT	637.84 ± 16.35^e^ (100)	ND	637.84 ± 16.35^e^
	YNQJ-RPT	414.43 ± 21.26^g^ (100)	ND	414.43 ± 21.26^g^
	GZQN-RPT	1067.30 ± 77.27^b^ (100)	ND	1067.3 ± 77.27^b^
	JXGZ-RPT	520.80 ± 3.79^f^ (100)	ND	520.80 ± 3.79^f^
mirificin	GDGZ-RPT	273.60 ± 13.00^b^ (100)	ND	273.60 ± 13.00^b^
	GDFS-RPT	94.66 ± 4.21^f^ (100)	ND	94.66 ± 4.21^f^
	GXWZ-RPT	206.28 ± 2.09^d^ (100)	ND	206.28 ± 2.09^d^
	GDSG-RPT	227.73 ± 15.50^c^ (100)	ND	227.73 ± 15.50^c^
	YNQJ-RPT	71.72 ± 1.03^g^ (100)	ND	71.72 ± 1.03^g^
	GZQN-RPT	409.60 ± 7.79^a^ (100)	ND	409.60 ± 7.79^a^
	JXGZ-RPT	125.98 ± 12.04^e^ (100)	ND	125.98 ± 12.04^e^
3′-methoxypuerarin	GDGZ-RPT	395.11 ± 8.31^a^ (100)	ND	395.11 ± 8.31^a^
	GDFS-RPT	89.94 ± 1.08^d^ (100)	ND	89.94 ± 1.08^d^
	GXWZ-RPT	231.54 ± 1.27^b^ (100)	ND	231.54 ± 1.27^b^
	GDSG-RPT	59.85 ± 5.26^e^ (100)	ND	59.85 ± 5.26^e^
	YNQJ-RPT	65.19 ± 3.00^e^ (100)	ND	65.19 ± 3.00^e^
	GZQN-RPT	103.11 ± 3.06^c^ (100)	ND	103.11 ± 3.06^c^
	JXGZ-RPT	60.01 ± 5.22^e^ (100)	ND	60.01 ± 5.22^e^
daidzin	GDGZ-RPT	598.09 ± 7.56^b^ (90.83)	60.35 ± 1.55^c^ (9.17)	658.44 ± 6.33^b^
	GDFS-RPT	555.11 ± 7.02^b^ (87.90)	76.42 ± 0.96^ab^ (12.10)	631.53 ± 7.79^b^
	GXWZ-RPT	602.61 ± 7.62^b^ (88.32)	79.73 ± 2.08^a^ (11.68)	682.34 ± 9.50^b^
	GDSG-RPT	413.06 ± 9.51^c^ (86.07)	66.85 ± 5.10^c^ (13.93)	479.90 ± 10.38^c^
	YNQJ-RPT	39.26 ± 3.94^e^ (37.83)	64.52 ± 1.61^c^ (62.17)	103.77 ± 4.00^e^
	GZQN-RPT	703.00 ± 43.79^a^ (91.81)	62.73 ± 5.06^c^ (8.19)	765.73 ± 46.36^a^
	JXGZ-RPT	295.84 ± 50.15^d^ (81.32)	67.95 ± 4.80^bc^ (18.68)	363.79 ± 50.08^d^
rutin	GDGZ-RPT	108.63 ± 2.29^a^ (100)	ND	108.63 ± 2.29^a^
	GDFS-RPT	52.80 ± 1.11^c^ (100)	ND	52.80 ± 1.11^c^
	GXWZ-RPT	62.99 ± 1.33^b^ (100)	ND	62.99 ± 1.33^b^
	GDSG-RPT	33.72 ± 4.01^d^ (100)	ND	33.72 ± 4.01^d^
	YNQJ-RPT	31.01 ± 2.27^d^ (100)	ND	31.01 ± 2.27^d^
	GZQN-RPT	34.40 ± 1.27^d^ (100)	ND	34.40 ± 1.27^d^
	JXGZ-RPT	33.45 ± 0.63^d^ (100)	ND	33.45 ± 0.63^d^
ferulic acid	GDGZ-RPT	288.76 ± 8.85^b^ (100)	ND	288.76 ± 8.85^b^
	GDFS-RPT	360.78 ± 11.06^a^ (100)	ND	360.78 ± 11.06^a^
	GXWZ-RPT	263.79 ± 8.09^c^ (100)	ND	263.79 ± 8.09^c^
	GDSG-RPT	91.67 ± 9.20^f^ (100)	ND	91.67 ± 9.20^f^
	YNQJ-RPT	137.51 ± 8.04^e^ (100)	ND	137.51 ± 8.04^e^
	GZQN-RPT	163.95 ± 2.63^d^ (100)	ND	163.95 ± 2.63^d^
	JXGZ-RPT	130.27 ± 13.81^e^ (100)	ND	130.27 ± 13.81^e^
genistin	GDGZ-RPT	709.86 ± 7.99^a^ (100)	ND	709.86 ± 7.99^a^
	GDFS-RPT	360.21 ± 7.58^c^ (100)	ND	360.21 ± 7.58^c^
	GXWZ-RPT	366.19 ± 0.55^c^ (100)	ND	366.19 ± 0.55^c^
	GDSG-RPT	86.82 ± 1.31^f^ (100)	ND	86.82 ± 1.31^f^
	YNQJ-RPT	211.43 ± 6.79^e^ (100)	ND	211.43 ± 6.79^e^
	GZQN-RPT	463.98 ± 19.47^b^ (100)	ND	463.98 ± 19.47^b^
	JXGZ-RPT	331.08 ± 4.66^d^ (100)	ND	331.08 ± 4.66^d^
quercitrin	GDGZ-RPT	222.41 ± 4.95^a^ (100)	ND	222.41 ± 4.95^a^
	GDFS-RPT	56.03 ± 1.25^c^ (100)	ND	56.03 ± 1.25^c^
	GXWZ-RPT	166.92 ± 3.72^b^ (100)	ND	166.92 ± 3.72^b^
	GDSG-RPT	42.56 ± 0.95^d^ (100)	ND	42.56 ± 0.95^d^
	YNQJ-RPT	32.44 ± 0.20^e^ (100)	ND	32.44 ± 0.20^e^
	GZQN-RPT	6.65 ± 0.11^g^ (100)	ND	6.65 ± 0.11^g^
	JXGZ-RPT	20.28 ± 2.07^f^ (100)	ND	20.28 ± 2.07^f^
phlorizin	GDGZ-RPT	284.69 ± 6.01^a^ (100)	ND	284.69 ± 6.01^a^
	GDFS-RPT	ND	ND	ND
	GXWZ-RPT	163.93 ± 3.46^b^ (100)	ND	163.93 ± 3.46^b^
	GDSG-RPT	11.91 ± 0.93^e^ (100)	ND	11.91 ± 0.93^e^
	YNQJ-RPT	35.21 ± 4.78^d^ (100)	ND	35.21 ± 4.78^d^
	GZQN-RPT	80.19 ± 9.28^c^ (100)	ND	80.19 ± 9.28^c^
	JXGZ-RPT	14.24 ± 1.02^e^ (100)	ND	14.24 ± 1.02^e^
ononin	GDGZ-RPT	363.10 ± 9.09^b^ (100)	ND	363.10 ± 9.09^b^
	GDFS-RPT	275.53 ± 5.80^c^ (100)	ND	275.53 ± 5.80^c^
	GXWZ-RPT	431.76 ± 9.08^a^ (100)	ND	431.76 ± 9.08^a^
	GDSG-RPT	181.17 ± 3.81^d^ (100)	ND	181.17 ± 3.81^d^
	YNQJ-RPT	177.25 ± 3.73^d^ (100)	ND	177.25 ± 3.73^d^
	GZQN-RPT	284.21 ± 5.98^c^ (100)	ND	284.21 ± 5.98^c^
	JXGZ-RPT	ND	ND	ND
daidzein	GDGZ-RPT	116.80 ± 2.78^c^ (93.98)	7.29 ± 0.19^d^ (6.02)	124.09 ± 2.62^c^
	GDFS-RPT	93.49 ± 2.22^d^ (91.88)	8.04 ± 0.21^d^ (8.12)	101.53 ± 2.06^d^
	GXWZ-RPT	53.07 ± 1.26^f^ (76.59)	15.8 ± 0.32^c^ (23.41)	68.87 ± 1.00^f^
	GDSG-RPT	9.57 ± 0.69^g^ (31.40)	15.71 ± 1.63^c^ (68.60)	25.28 ± 1.74^g^
	YNQJ-RPT	65.39 ± 1.91^e^ (79.75)	17.53 ± 0.73^bc^ (20.25)	82.91 ± 2.51^e^
	GZQN-RPT	171.49 ± 7.14^a^ (89.88)	21.72 ± 1.81^ab^ (10.12)	193.21 ± 7.30^a^
	JXGZ-RPT	130.29 ± 9.04^b^ (86.86)	23.14 ± 2.98^a^ (13.14)	153.43 ± 8.77^b^
genistein	GDGZ-RPT	30.89 ± 0.85^a^ (100)	ND	30.89 ± 0.85^a^
	GDFS-RPT	12.54 ± 1.80^d^ (100)	ND	12.54 ± 1.80^d^
	GXWZ-RPT	21.40 ± 0.59^bc^ (100)	ND	21.40 ± 0.59^bc^
	GDSG-RPT	18.25 ± 0.60^c^ (100)	ND	18.25 ± 0.60^c^
	YNQJ-RPT	18.91 ± 1.40^c^ (100)	ND	18.91 ± 1.40^c^
	GZQN-RPT	24.27 ± 0.59^b^ (100)	ND	24.27 ± 0.59^b^
	JXGZ-RPT	23.67 ± 2.95^b^ (100)	ND	23.67 ± 2.95^b^
puerarin derivative A	GDGZ-RPT	ND	491.92 ± 16.34^b^ (100)	491.92 ± 16.34^b^
	GDFS-RPT	ND	350.21 ± 10.49^c^ (100)	350.21 ± 10.49^c^
	GXWZ-RPT	ND	323.36 ± 22.54^c^ (100)	323.36 ± 22.54^c^
	GDSG-RPT	ND	148.49 ± 3.27^d^ (100)	148.49 ± 3.27^d^
	YNQJ-RPT	ND	168.97 ± 18.82^d^ (100)	168.97 ± 18.82^d^
	GZQN-RPT	ND	930.29 ± 52.54^a^ (100)	930.29 ± 52.54^a^
	JXGZ-RPT	ND	63.59 ± 3.90^e^ (100)	63.59 ± 3.90^e^
*p*-coumaric acid	GDGZ-RPT	ND	11.63 ± 1.48^a^ (100)	11.63 ± 1.48^a^
	GDFS-RPT	ND	11.96 ± 1.52^a^ (100)	11.96 ± 1.52^a^
	GXWZ-RPT	ND	7.64 ± 0.97^b^ (100)	7.64 ± 0.97^b^
	GDSG-RPT	ND	1.58 ± 0.29^cd^ (100)	1.58 ± 0.29^cd^
	YNQJ-RPT	ND	1.43 ± 0.02^d^ (100)	1.43 ± 0.02^d^
	GZQN-RPT	ND	6.74 ± 0.48^b^ (100)	6.74 ± 0.48^b^
	JXGZ-RPT	ND	3.84 ± 0.10^c^ (100)	3.84 ± 0.10^c^

RPT: *radix puerariae thomsonii*. ND: not detected. ^⁎^Values with no common letters in each column are significantly different (*p* < 0.05). Data are showed as mean ± SD (*n* = 3). ^⁎⁎^Percentage contributions to the total content are showed in parentheses. The contents of puerarin derivative A are expressed as the equivalent of puerarin.

The compositions of phenolic compounds were generally similar among different RPT cultivars, except that phlorizin was not detected in GDFS-RPT and ononin was not detected in JXGZ-RPT. However, the contents of the detected compounds varied significantly. Puerarin was the most abundant compound among all detected phenolics in RPTs, ranging from 414.43 to 1340.6 μg/g DW, with an average total content of 809.83 μg/g DW. GDGZ-RPT had the highest puerarin content, followed by GZQN-RPT and GXWZ-RPT, and YNQJ-RPT had the least puerarin. Among the 5 puerarin derivatives, puerarin derivative A had a higher content than the other 4 compounds, with an average of 353.83 μg/g DW. Puerarin derivative A was quantified using puerarin as the standard compound due to the lack of commercially available standards. Puerarin and its derivatives were found in free form in RPTs, except for puerarin derivative A, which was detected in bound form. The only 2 isoflavones detected in both free and bound forms in RPTs were daidzin and its aglycone, daidzein. Daidzin was the second most abundant compound in RPTs among the 16 detected phenolics, with an average total content of 526.50 μg/g DW. Free daidzin contributed to >80% of the total content in all the determined RPTs, except for YNQJ-RPT, where free daidzin accounted for only 37.83%. The total contents of daidzein, ranging from 25.28 to 193.21 μg/g DW, were much lower than its glycoside, daidzin. Genistin was the third most abundant phenolic compound in RPTs, with an average content of 361.37 μg/g DW. Similarly, the contents of genistin were much higher than those of its aglycone, genistein. *Leguminous* plants, especially soybeans, are the main source of isoflavones, the contents of predominant isoflavone daidzin in different legumes cultivars varied greatly in published data, ranging from 8.7 to 4466.4 μg/g DW (Leuner et al., 2013). Puerarin is the key component of RP used for evaluating the quality of RPT or RPL (Zhang, Lam, & Zuo, 2013). Due to the significant bioactivities of daidzin and genistin, their contents were also considered as an index of reference for evaluating RP (Cherdshewasart, Subtang, & Dahlan, 2007).

The 16 compounds detected in RPTs could be categorized into 3 groups: isoflavones, flavones, and phenolic acids. The subtotal contents of each category are presented in Table 2. Among the 3 groups of phenolics, isoflavones showed the highest content in RPTs. The free, bound, and total isoflavone contents ranged from 1183.79 (YNQJ-RPT) to 4404.06 μg/g DW (GDGZ-RPT), 154.69 (JXGZ-RPT) to 1014.74 μg/g DW (GZQN-RPT), and 1434.81 (YNQJ-RPT) to 4938.40 μg/g DW (GZQN-RPT), respectively. Free isoflavone accounted for 79.45% to 91.74% of the total content. Flavones were only detected in free form, ranging from 67.96 μg/g DW (JXGZ-RPT) to 615.72 μg/g DW (GDGZ-RPT). The total contents of phenolic acids ranged from 93.25 (GDSG-RPT) to 372.74 μg/g DW (GDFS-RPT), with free form compounds contributing 96.05% to 98.97%. In terms of overall quantity, GDGZ-RPT had the highest polyphenol content (5519.72 μg/g DW), followed by GZQN-RPT (5228.67 μg/g DW), and GXWZ-RPT (4204.88 μg/g DW).

**Table 2:** Subtotal of different kinds of phenolic compounds in RPTs from different growing regions (μg/g dry weight).

Polyphenols	RPTs	Free	Bound	Total
isoflavones	GDGZ-RPT	4044.06 ± 119.92^a ⁎^(87.85)^⁎⁎^	559.56 ± 15.68^b^ (12.15)	4603.62 ± 129.20^b^
	GDFS-RPT	2477.31 ± 33.95^c^ (85.07)	434.67 ± 9.82^c^ (14.93)	2911.98 ± 29.59^d^
	GXWZ-RPT	3120.73 ± 26.46^b^ (88.17)	418.89 ± 24.40^c^ (11.83)	3539.62 ± 43.23^c^
	GDSG-RPT	2035.73 ± 18.26^d^ (89.81)	231.05 ± 8.66^d^ (10.19)	2266.78 ± 10.91^e^
	YNQJ-RPT	1183.79 ± 26.14^f^ (82.51)	251.02 ± 19.33^d^ (17.49)	1434.81 ± 7.08^g^
	GZQN-RPT	3923.66 ± 118.07^a^ (79.45)	1014.74 ± 55.28^a^ (20.55)	4938.40 ± 173.24^a^
	JXGZ-RPT	1718.65 ± 54.97^e^ (91.74)	154.69 ± 1.29^e^ (8.26)	1873.33 ± 54.49^f^
flavones	GDGZ-RPT	615.72 ± 13.18^a^ (100)	ND	615.72 ± 13.18^a^
	GDFS-RPT	108.83 ± 2.35^cd^ (100)	ND	108.83 ± 2.35^cd^
	GXWZ-RPT	393.83 ± 8.46^b^ (100)	ND	393.83 ± 8.46^b^
	GDSG-RPT	88.18 ± 3.73^e^ (100)	ND	88.18 ± 3.73^e^
	YNQJ-RPT	98.66 ± 6.67^de^ (100)	ND	98.66 ± 6.67^de^
	GZQN-RPT	119.59 ± 6.91^c^ (100)	ND	119.59 ± 6.91^c^
	JXGZ-RPT	67.96 ± 0.52^f^ (100)	ND	67.96 ± 0.52^f^
phenolic acids	GDGZ-RPT	288.76 ± 8.85^b^ (96.13)	11.63 ± 1.48^a^ (3.87)	300.38 ± 8.66^b^
	GDFS-RPT	360.78 ± 11.06^a^ (96.79)	11.96 ± 1.52^a^ (3.21)	372.74 ± 10.84^a^
	GXWZ-RPT	263.79 ± 8.09^c^ (97.19)	7.64 ± 0.97^b^ (2.81)	271.43 ± 7.94^c^
	GDSG-RPT	91.67 ± 9.20^f^ (98.31)	1.58 ± 0.29^cd^ (1.69)	93.25 ± 9.06^f^
	YNQJ-RPT	137.51 ± 8.04^e^ (98.97)	1.43 ± 0.02^d^ (1.03)	138.93 ± 8.04^e^
	GZQN-RPT	163.95 ± 2.63^d^ (96.05)	6.74 ± 0.48^b^ (3.95)	170.69 ± 3.06^d^
	JXGZ-RPT	130.27 ± 13.81^e^ (97.14)	3.84 ± 0.10^c^ (2.86)	134.10 ± 13.73^e^
sum	GDGZ-RPT	4948.54 ± 139.50^a^ (89.65)	571.18 ± 14.33^b^ (10.35)	5519.72 ± 146.68^a^
	GDFS-RPT	2946.92 ± 43.69^d^ (86.84)	446.63 ± 10.22^c^ (13.16)	3393.55 ± 35.74^d^
	GXWZ-RPT	3778.35 ± 40.44^c^ (89.86)	426.53 ± 23.64^c^ (10.14)	4204.88 ± 50.87^c^
	GDSG-RPT	2215.58 ± 19.33^e^ (90.50)	232.63 ± 8.94^d^ (9.50)	2448.20 ± 10.52^e^
	YNQJ-RPT	1419.96 ± 30.00^g^ (84.91)	252.44 ± 19.35^d^ (15.09)	1672.40 ± 10.90^g^
	GZQN-RPT	4207.19 ± 113.71^b^ (80.46)	1021.48 ± 55.61^a^ (19.54)	5228.67 ± 169.24^b^
	JXGZ-RPT	1916.88 ± 50.16^f^ (92.36)	158.52 ± 1.37^e^ (7.64)	2075.40 ± 49.37^f^

RPT: *radix puerariae thomsonii*. ND: not detected. ^⁎^Values with no common letters in each column are significantly different (*p* < 0.05). Data are showed as mean ± SD (*n* = 3). ^⁎⁎^Percentage contributions to the total content are showed in parentheses.

In the present study, 4 phenolic compounds, daidzin, daidzein, puerarin derivative A, and *p*-coumaric acid were identified in the bound fractions of RPTs, among of which, puerarin derivative A and *p*-coumaric acid were only detected in bound form. Puerarin derivative A was the predominant bound phenolic compound in RPTs. Daidzin and daidzein have been detected in RPT as free form. *p*-Coumaric acid is an important phenolic compound occurring in bound form in grains, fruits, and vegetables (Feng et al., 2021). This study reported for the first time that these compounds occurred in RPTs in bound form. As mentioned above, phenolics, especially isoflavones, were key compounds for evaluating the quality of RPT. Therefore, the detection of bound phenolics in our study undoubtedly expanded the understanding of the active components presented in RPT.

### 3.3 Antioxidant activities of RPTs

The ORAC and PSC results of free and bound fractions of RPT from 7 growing regions are shown in Table 3. Both ORAC and PSC results displayed potential antioxidant properties. The total ORAC values ranged from 49.75 (JXGZ-RPT) to 124.23 μmol TE/g DW (GZQN-RPT), with an average of 83.72 μmol TE/g DW. The *CV* for the total ORAC value was 28.38%, indicating a significant regional difference in the ORAC antioxidant activity of RPTs. The free fractions of RPT exhibited ORAC values ranging from 33.07 (JXGZ-RPT) to 75.07 μmol TE/g DW (GZQN-RPT), accounting for 60.43% (GZQN-RPT) to 71.85% (GDSG-RPT) to the total antioxidant activity. The total PSC values ranged from 1.26 (JXGZ-RPT) to 8.48 (GXWZ-RPT) μmol TE/g DW, with an average of 4.97 μmol TE/g DW. The *CV* of the total PSC value was 49.23%, indicating that the PSC activity demonstrated more cultivar variation compared to the ORAC activity in RPTs. The percentage contributions of the free fraction to the total PSC value were from 29.16% (JXGZ-RPT) to 55.45% (GDGZ-RPT). The results indicated that bound fractions contributed a lot to the total antioxidant activity of RPTs. Furthermore, the bound fractions contributed more to the total PSC activity than ORAC in all RPTs. This difference could be attributed to the variation of antioxidant capacity of phenolic compounds when determined by different methods. As reported in the research of Feng et al. (2021), the ORAC value of vanillic acid (1.88 μmol TE/μmol) was 61.7 times higher than its PSC value (0.03 μmol TE/μmol), while the ORAC value of thomasidioic acid (3.99 μmol TE/μmol) was lower than its PSC value (5.98 μmol TE/μmol).

**Table 3:** Antioxidant activity of the free and bound phenolic of RPTs and their percentage contributions to the total (μmol TE/g dry weight).

RPTs	ORAC			PSC		
	Free	Bound	Total	Free	Bound	Total
GDGZ-RPT	61.39 ± 1.00^b⁎^ (65.53)^⁎⁎^	32.29 ± 1.52^c^ (34.47)	93.67 ± 2.52^b^	3.91 ± 0.52^a^ (55.45)	3.14 ± 0.26^b^ (44.55)	7.05 ± 0.71^ab^
GDFS-RPT	51.05 ± 0.56^c^ (63.50)	29.34 ± 1.04^d^ (36.50)	80.39 ± 0.49^d^	2.77 ± 0.37^b^ (49.84)	2.79 ± 0.23^b^ (50.16)	5.55 ± 0.59^b^
GXWZ-RPT	59.02 ± 0.21^b^ (61.51)	36.93 ± 1.32^b^ (38.49)	95.95 ± 1.26^b^	3.57 ± 0.06^ab^ (42.14)	4.91 ± 0.04^a^ (57.86)	8.48 ± 0.05^a^
GDSG-RPT	39.11 ± 1.67^d^ (71.85)	15.32 ± 0.40^f^ (28.15)	54.43 ± 2.06^e^	1.37 ± 0.08^c^ (47.12)	1.54 ± 0.19^c^ (52.88)	2.91 ± 0.27^c^
YNQJ-RPT	60.71 ± 0.76^b^ (69.29)	26.91 ± 1.38^e^ (30.71)	87.62 ± 2.04^c^	1.48 ± 0.18^c^ (50.85)	1.43 ± 0.30^c^ (49.15)	2.92 ± 0.44^c^
GZQN-RPT	75.07 ± 0.48^a^ (60.43)	49.16 ± 1.13^a^ (39.57)	124.23 ± 0.81^a^	3.12 ± 0.62^ab^ (47.27)	3.48 ± 0.49^b^ (52.73)	6.60 ± 1.11^b^
JXGZ-RPT	33.07 ± 2.10^e^ (66.46)	16.68 ± 0.44^f^ (33.54)	49.75 ± 2.53^f^	0.37 ± 0.05^d^ (29.16)	0.89 ± 0.06^c^ (70.84)	1.26 ± 0.11^d^

ORAC: oxygen radical scavenging capacity; PSC: peroxyl radical scavenging capacity; TE: Trolox equivalents; RPT: *radix puerariae thomsonii*; ^⁎^Values with no common letters in the same column are significantly different, *p* < 0.05. ^⁎⁎^Values in parentheses indicate the percentage contribution to the total content. Data was showed as mean ± SD (*n* = 3).

The average total ORAC value of the 7 RPTs was 31.67 μmol TE/g FW when the moisture contents in RPTs (56.93–65.74%) were taken into consideration. The ORAC antioxidant activity of RPTs was higher than those of most common vegetables, such as asparagus (30.17 μmol TE/g FW), beets (27.74 μmol TE/g FW), broccoli (15.90 μmol TE/g FW), cabbages (13.59 μmol TE/g FW), and carrots (12.15 μmol TE/g FW), but lower than those of black beans (75.93 μmol TE/g DW) and red kidney beans (144.04 μmol TE/g DW) (Wu et al., 2004). The average total PSC value of RPTs (4.97 μmol TE/g DW) exceeded that of the common whole grains, such as rice (2.95 μmol TE/g DW), oats (3.69 μmol TE/g DW), and wheat (3.87 μmol TE/g DW) (Adom & Liu, 2005). The results indicated that RPTs had significant antioxidant activity.

### 3.4 ADH and ALDH activation activities of RPTs

**Fig. 3:**
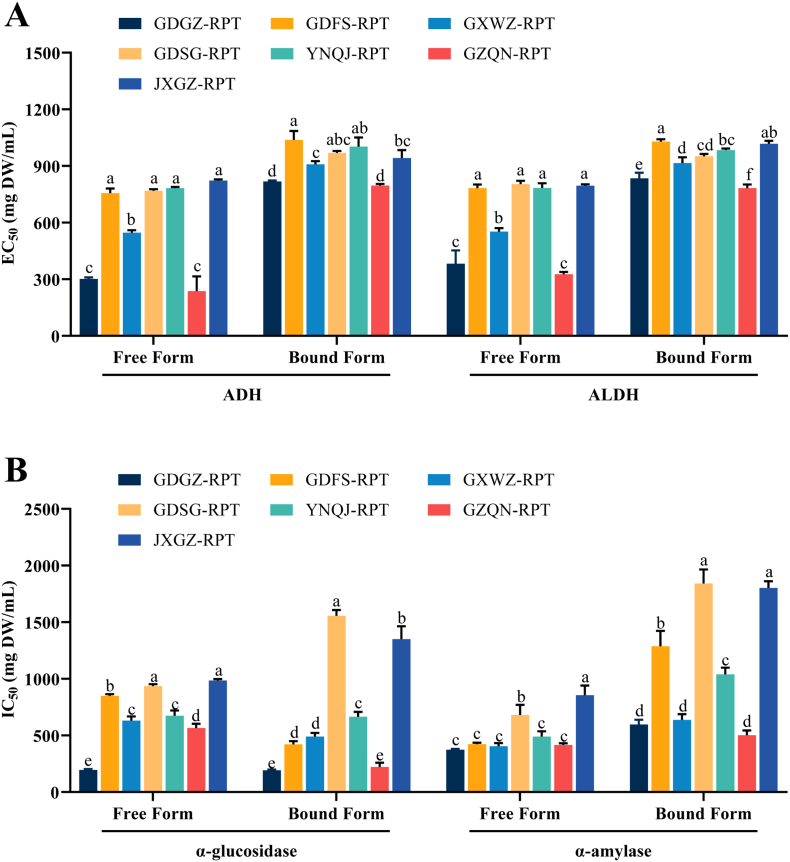
Activating effects on ADH, ALDH and inhibition to α-glucosidase and α-amylase by free and bound phenolics of RPTs from different growing regions. ADH: alcohol dehydrogenase; ALDH: aldehyde dehydrogenase; RPT: *radix puerariae thomsonii*. DW: dry weight. *Bars with no common letters in the same group are significantly different (*p* < 0.05). Data are showed as mean ± SD (*n* = 3).

ADH and ALDH are crucial enzymes in alcohol metabolism. The activating effects of RPT on ADH and ALDH are presented in Fig. 3A and expressed as EC_50_. Both free and bound fractions of RPTs demonstrated significant activating effects on ADH and ALDH in a dose-dependent manner. The EC_50_ values for ADH activation ranged from 236.93 to 822.35 mg DW/mL for the free fraction, and from 796.76 to 1037.67 mg DW/mL for bound fraction. The free fraction of GZQN-RPT and GDGZ-RPT displayed the lowest EC_50_ value followed by GXWZ-RPT. The bound fractions of other 4 RPTs exhibited the weakest activating effects with the highest EC_50_ values. For ALDH activation, the EC_50_ values of the free and bound fractions ranged from 326.67 to 803.87 mg DW/mL and 782.80 to 1029.57 mg DW/mL, respectively. Similarly, the free fractions of GZQN-RPT and GDGZ-RPT exhibited the highest activating effects, followed by GXWZ-RPT, and the other 4 RPTs showed the lowest activity. In terms of the bound fractions, GZQN-RPT demonstrated the highest ALDH activation activity, followed by GDGZ-RPT, GXWZ-RPT, and GDSG-RPT. In general, the cultivars GZQN-RPT and GDGZ-RPT had much higher activating effects on the alcohol metabolizing enzymes than the other 5 RPT cultivars, especially for the free fraction.

The elimination of alcohol from the body mainly relies on the alcohol metabolizing enzymes ADH and ALDH in the liver. ADH first converts alcohol into acetaldehyde, which is then metabolized by ALDH into carbon dioxide and water. Certain isoflavone compounds were proven to speed up alcohol metabolism and decrease the accumulation of acetaldehyde by modulating the activities of alcohol metabolizing enzymes in the liver (Keung & Vallee, 1998). Choi et al. (2009) reported that Sophoronol 2, an isoflavone compound, increased the activities of ADH and ALDH by up to 9 folds *in vitro* at concentrations of 100 and 50 μg/mL, respectively. Our study also showed that free and bound phenolic extracts of RPTs from 7 different growing regions could activate ADH and ALDH. Water extracts of RPT and RPL, containing puerarin, daidzin, and daidzein, were also effective on enhancing the activities of liver ADH and ALDH, improving alcohol metabolism in rats treated with alcohol (Yang et al., 2023). These extracts could also mitigate the adverse effects of alcohol intake by enhancing lipid metabolism and the hepatic antioxidant defense system (Lee, 2004). Excessive alcohol consumption, surpassing the metabolic capacities of ADH and ALDH, would trigger the microsomal ethanol oxidation system (MEOS), resulting in oxidative stress. The isoflavones of RPT, such as puerarin and genistin, could intervene in the activity of CYP2E1 in the MEOS, and enhance the activities of superoxide dismutase and catalase, and alleviating liver injuries induced by oxidative stress (Chen et al., 2013).

### 3.5 α-Glucosidase and α-amylase inhibitory activities of RPTs

The inhibitory activities of RPT on α-glucosidase are presented in Fig. 3B and expressed as IC_50_ values. Both free and bound fractions demonstrated a concentration-dependent inhibition of α-glucosidase activity. The IC_50_ values of free fractions were from 195.50 to 986.02 mg DW/mL. GDGZ-RPT exhibited the highest inhibitory activity, followed by GZQN-RPT, GXWZ-RPT, and YNQJ-RPT. JXGZ-RPT and GDSG-RPT had similar inhibitory activity (*p* > 0.05), lower than the other 5 cultivars (*p* < 0.05). The IC_50_ values of the bound fractions showed a big variation, ranging from 192.80 to 1556.46 mg DW/mL. GDGZ-RPT and GZQN-RPT displayed the strongest inhibitory ability, followed by GDFS-RPT and GXWZ-RPT. GDSG-RPT and JXGZ-RPT showed far lower inhibitory activity than the other 5 cultivars. The IC_50_ values for α-amylase of the free fractions ranged from 373.63 to 855.74 mg DW/mL. GDGZ-RPT, GXWZ-RPT, GZQN-RPT, GDFS-RPT, and YNQJ-RPT displayed equivalent inhibitory activity (*p* > 0.05), a little bit higher than the other 2 cultivars, GDSG-RPT and JXGZ-RPT (*p* < 0.05). The bound fractions also showed wide range in inhibition of α-amylase, with IC_50_ values ranging from 501.85 to 1840.95 mg DW/mL. GZQN-RPT, GDGZ-RPT, and GXWZ-RPT displayed stronger inhibitory activity than the other cultivars. GDSG-RPT and JXGZ-RPT showed the weakest activity with similar IC_50_ values (*p* > 0.05).

Tadera et al. (2006) found that isoflavones showed the strongest inhibitory effects on α-amylase and α-glucosidase among 16 flavonoid compounds. The robust inhibitory effect of isoflavones on the carbohydrate hydrolyzing enzymes could be attributed to the presence of hydroxyl groups on the aromatic rings A and B of its skeleton (Xiao, Ni, Kai, & Chen, 2013). Specifically, the hydroxyl groups at the C-5 and C-7 positions of ring A played a crucial role in binding with the active sites of the enzymes (Xiao, Kai, Yamamoto, & Chen, 2013). Wang et al. (2017) discovered that phenolic compounds derived from RPL, including daidzein, puerarin, ononin, *3S, 4R*-tuberosin, and loboflavate exhibited intensive inhibitory activities against α-glucosidase. The IC_50_ values of these compounds were 23.01, 524.08, 422.89, 378.89, and 1.79 μM, respectively, which were more potent than acarbose (1998.79 μM), a standard α-glucosidase inhibitor. Our study revealed that both free and bound fractions of the cultivars GZQN-RPT and GDGZ-RPT showed stronger inhibitory effects on the carbohydrate hydrolyzing enzymes than the other cultivars. The higher contents of isoflavones bearing hydroxyl groups at C-5 or C-7 on ring A, including puerarin, mirificin, 3′-methoxypuerarin, genistin, daidzein, and genistein, in GZQN-RPT and GDGZ-RPT than in other cultivars might account for their advantage in enzyme inhibiting activities over the other cultivars.

Inhibiting the intestinal carbohydrate hydrolyzing enzymes was an important approach in the management of type 2 diabetes, which thereby prevented the rapid increase of postprandial glucose levels. The inhibitory activities of RPTs against α-amylase and α-glucosidase indicated that they might be beneficial in the prevention and treatment of diabetes. A significant reduction in fasting blood glucose and improved glucose and insulin tolerance were observed in ob/ob mice that were fed with RPL extract (0.2%, *w/w*, in the diet) primarily containing puerarin after 8-month intervention (Prasain, Peng, Rajbhandari, & Wyss, 2012). Despite the lack of direct evidence on the hypoglycemic effects of RPT, it is suggested that RPTs, especially the cultivars GZQN-RPT and GDGZ-RPT, might have potential to help maintain glucose metabolism homeostasis, considering their significant inhibitory effects on the carbohydrate hydrolyzing enzymes and their similar phenolic composition to RPL.

## 4 Conclusion

The total contents of phenolics and flavonoids showed significant variations among 7 RPT cultivars from different growing regions, with the ranges of 148.71–435.32 mg GAE/100 g DW and 561.93–826.11 mg CE/100 g DW, respectively. GDGZ-RPT exhibited higher TPC and TFC compared to the other RPT cultivars. Bound phenolics were firstly determined in RPTs in the present study, accounted for 20.64–38.28% of the total phenolic contents and 32.77–47.29% of the total flavonoid contents. Sixteen phenolic compounds were detected in different RPTs, including 11 isoflavones, 3 flavones, and 2 phenolic acids, among which puerarin had the highest content. Daidzin, daidzein, puerarin derivative A and *p*-coumaric acid were found to occur in bound form in RPTs, and the latter 2 compounds were only detected in bound fractions. Furthermore, a quantitative analysis of bound phenolic compounds in RPT was conducted for the first time and found that puerarin derivative A was the major component, while bound daidzin, daidzein, and *p*-coumarin accounted for a smaller proportion. The antioxidant activity of 7 RPT cultivars, determined by ORAC and PSC, exhibited significant cultivar differences with *CVs* of 28.38% and 49.23%, respectively. Additionally, the regulatory activities of enzymes, including alcohol metabolizing enzymes ADH and ALDH, and carbohydrate hydrolyzing enzymes α-glucosidase and α-amylase, were significantly different among 7 RPT cultivars. The cultivars GZQN-RPT and GDGZ-RPT showed much higher regulatory effects on the enzymes than the other cultivars. The bound fractions showed equivalent effects on the enzymes as the free fractions. RPTs, especially the cultivars GZQN-RPT and GDGZ-RPT, had abundant phytochemicals with the potentials for antioxidation, enhancing alcohol metabolism, and controlling fasting blood glucose. RPTs have the potential to be used in the manufacture of functional foods.

## CRediT authorship contribution statement

**Weixin Li:** Writing – original draft, Data curation, Formal analysis, Visualization. **Xuchao Jia:** Data curation. **Min Zhang:** Conceptualization, Funding acquisition, Supervision. **Yanxia Chen:** Investigation, Software. **Lihong Dong:** Methodology, Validation. **Fei Huang:** Funding acquisition, Validation. **Qin Ma:** Data curation, Validation. **Dong Zhao:** Data curation, Investigation. **Ruifen Zhang:** Data curation, Funding acquisition, Methodology, Project administration, Supervision, Validation, Writing – review & editing.

## Declaration of competing interest

The authors declare that they have no known competing financial interests or personal relationships that could have appeared to influence the work reported in this paper.

## Data Availability

Data will be made available on request.

## Appendix A Supplementary data

Supplementary material Image 1
