# Supplementary material for: Free and bound phenolic profiles of Radix Puerariae Thomsonii from different growing regions and their bioactivities
Source: Food Chem X. 2024 Apr 5;22:101355. doi: 10.1016/j.fochx.2024.101355 (PMC11043822; doi:10.1016/j.fochx.2024.101355)

**Supplementary data**

**Free and bound phenolic profiles of *Radix Puerariae Thomsonii* from different growing regions and their bioactivities**

Weixin Li ^a^, Mingwei Zhang ^b^, Xuchao Jia ^b^, Min Zhang ^a^, Yanxia Chen ^b^, Lihong Dong ^b^, Fei Huang ^b^, Qin Ma ^b^, Dong Zhao ^b^, Ruifen Zhang^b,^ *

*^a^* State Key Laboratory of Food Nutrition and Safety, College of Food Science and Engineering, Tianjin University of Science and Technology, Tianjin 300457, PR China

*^b^* Sericultural & Agri-Food Research Institute Guangdong Academy of Agricultural Sciences/Key Laboratory of Functional Foods, Ministry of Agriculture/Guangdong Key Laboratory of Agricultural Products Processing, Guangzhou 510610, PR China

**Figure captions:**

**Fig. S1.** Pictures of RPTs from 7 growing regions. GDGZ-RPT (Guangzhou, Guangdong), GDFS-RPT (Foshan, Guangdong), GXWZ-RPT (Wuzhou, Guangxi), GDSG-RPT (Shaoguan, Guangdong), YNQJ-RPT (Qujing, Yunnan), GZQN-RPT (Qiannan, Guizhou), JXGZ-RPT (Ganzhou, Jiangxi). RPT: *radix puerariae thomsonii*.

**Fig. S2.** Secondary mass spectrometry information maps of 16 compounds. A, 3′-hydroxypuerarin; B, 6″-*O*-xylosidepuerarin; C, puerarin; D, mirificin; E, 3′-methoxypuerarin; F, daidzin; G, rutin; H, ferulic acid; I, genistin; J, quercitrin; K, phlorizin; L, ononin; M, daidzein; N, genistein; O, puerarin derivative A; P, *p*-coumaric acid.

**Fig. S1.** Pictures of RPTs from 7 growing regions


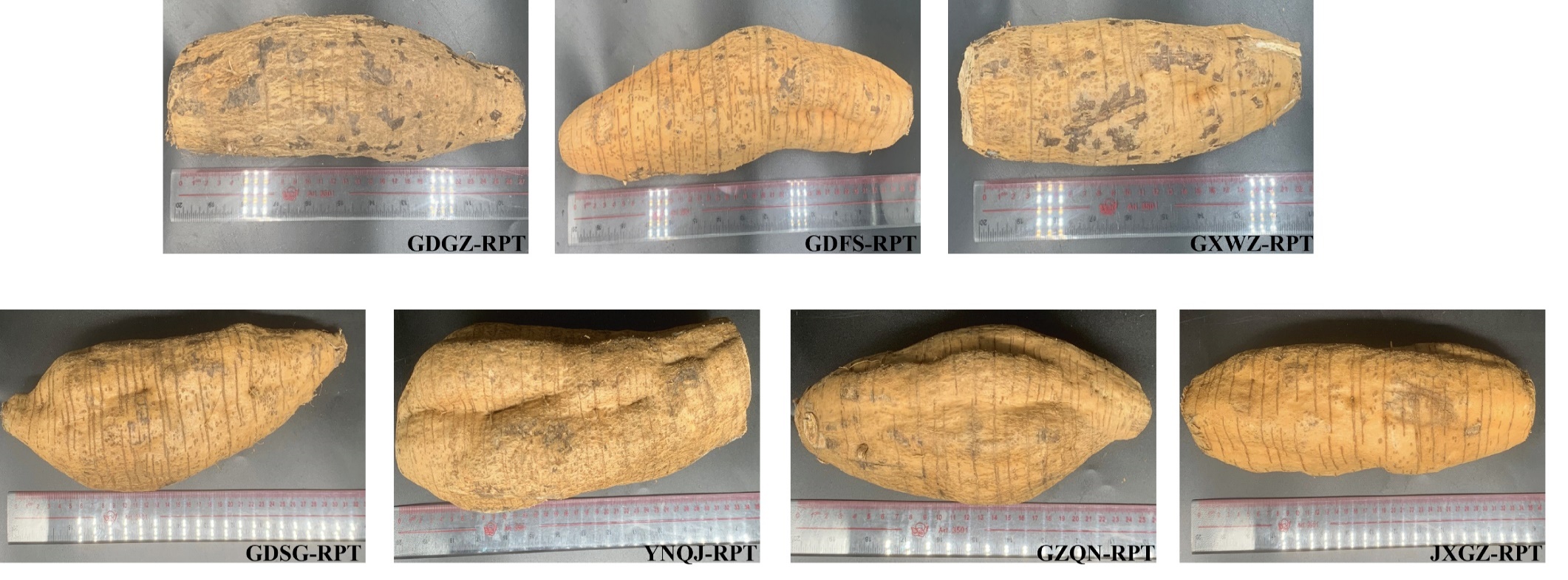


**Fig. S2.** Secondary mass spectrometry information maps of 16 compounds


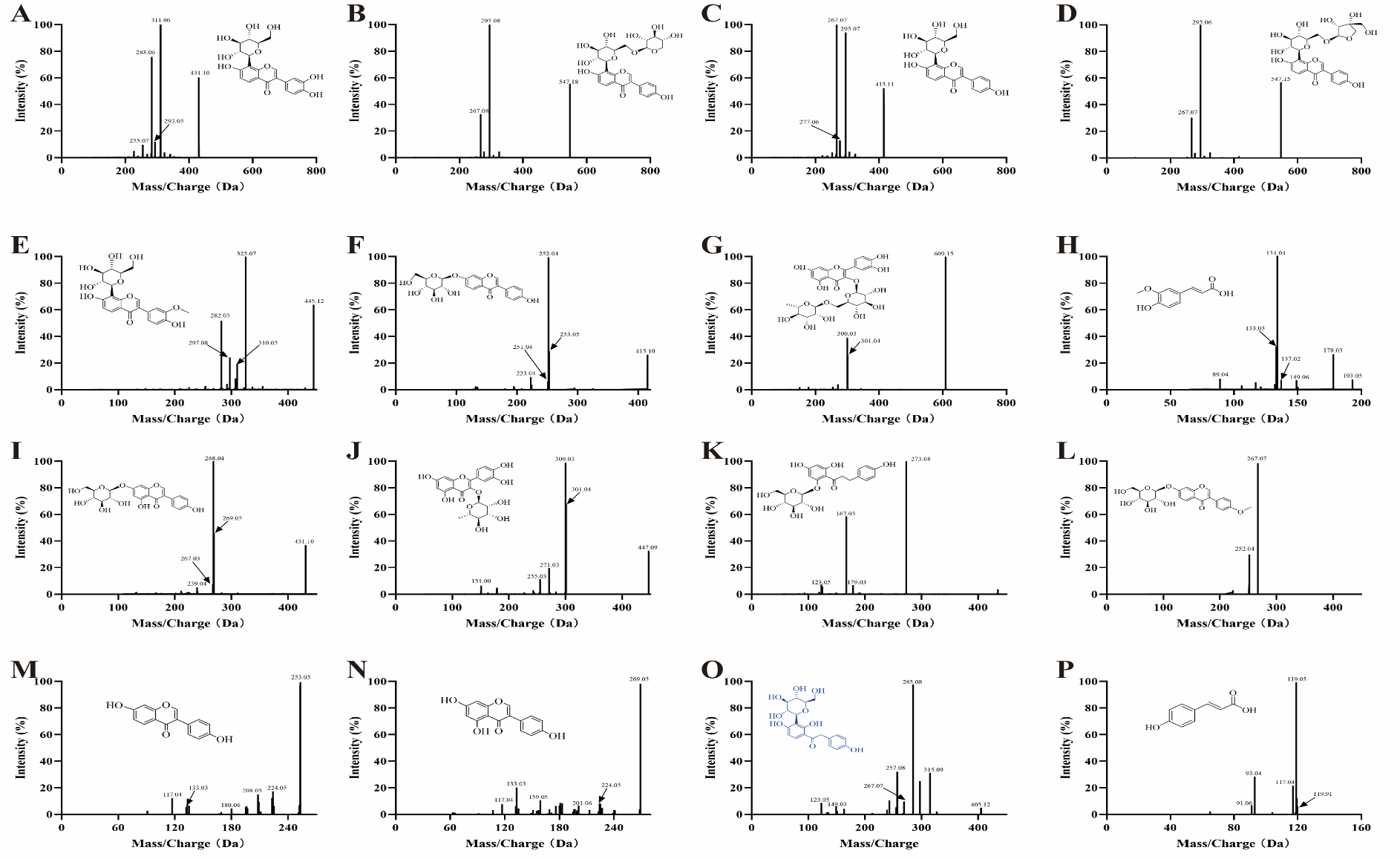

Supplement: Supplementary file 1 — Supplementary material [file mmc1.docx]
